# Dissection-related carotid-cavernous fistula (CCF) following surgical revascularization of chronic internal carotid artery occlusion: a new subtype of CCF and proposed management

**DOI:** 10.1186/s41016-019-0180-9

**Published:** 2020-01-10

**Authors:** Ao-Fei Liu, Chen Li, Wengui Yu, Li-Mei Lin, Han-Cheng Qiu, Yi-Qun Zhang, Xian-Li Lv, Kai Wang, Ce Liu, Wei-Jian Jiang

**Affiliations:** 1 Department of Vascular Neurosurgery, New Era Stroke Care and Research Institute, The PLA Rocket Force Characteristic Medical Center, No. 16 Xinjiekouwai Street, Xicheng District, Beijing, 100088 China; 2 Department of Neurology, University of California, Irvine, Irvine, CA USA; 3 Department of Neurosurgery, University of California, Irvine, Irvine, CA USA; 4 Department of Neurosurgery, Tsinghua Changgung Hospital of Tsinghua University, Beijing, China

**Keywords:** Arterial dissection, Carotid-cavernous fistula, Hybrid surgery, Internal carotid artery occlusion, Stenting

## Abstract

**Background:**

The development of carotid-cavernous fistulas (CCFs) during surgical recanalization of chronic internal carotid artery occlusion (ICAO) may be secondary to severe ICA dissection rather than a focal tear of the cavernous ICA seen in typical traumatic CCFs. The purpose of this study is to investigate the causal relationship between the CCFs and severe ICA dissections and to characterize technical outcomes after treatment with stenting.

**Methods:**

Five patients underwent treatment with self-expanding stents due to intraprocedural CCF and ICA dissection following surgical removal of ICAO plaque. The stents were telescopically placed via true channel of the dissection. Safety of the procedure was evaluated with 30-day stroke and death rate. Procedural success was determined by the efficacy of CCF obliteration and ICAO recanalization with angiography.

**Results:**

All CCFs were associated with spiral and long segmental dissection from the cervical to cavernous ICA. After stenting, successful dissection reconstruction with TICI 3 was achieved in all patients, with complete (*n* = 4) or partial CCF (*n* = 1) obliteration. No patient had CCF syndrome, stroke, or death during follow-up of 6 to 37 months; but one patient had pulsatile tinnitus, which resolved 1 year later. Angiography at 6 to 24 months demonstrated CCF obliteration in all 5 patients and durable ICA patency in 4 patients.

**Conclusions:**

Intraprocedural CCFs with spiral and cervical-to-cavernous ICA dissection during ICAO surgery are dissection-related because of successful obliteration after stenting for dissection reconstruction. Self-expanding stenting through true channel of the dissection, serving as implanting stent-autograft, may be an optimal therapy for the atypical CCF complication from ICAO surgery.

## Background

It is well known that traumatic carotid-cavernous fistulas (CCFs) are caused by blunt and penetrating head trauma, as well as iatrogenic injury from transarterial neurointervention, the use of Fogarty balloon catheter in carotid endarterectomy (CEA), percutaneous treatment of trigeminal neuralgia, or transsphenoidal resection of pituitary tumor [1–5]. In the majority of the cases, the fistula results from the direct connection between the cavernous internal carotid artery (ICA) and the cavernous sinus (CS), with a focal and transversal intima-to-adventitia tear [1–4]. Endovascular repair has recently become the mainstay therapy for definitive treatment of CCFs [5–8]. Available options include intracavernous sinus embolization with detachable balloons, coils, liquid embolic agents, or a combination of these via arterial and/or venous route; transarterial covered stenting across the fistulous connection; or intracavernous sinus embolization combined with transarterial flow-diverting stenting [5–10].

CCFs can also develop as a complication of surgical recanalization of symptomatic chronic internal carotid artery occlusions (ICAOs) [11, 12]. In these cases, the CCFs are observed on angiography after immediate open surgical removal of the ICAO plaque and are likely associated with severe dissection from the cervical to cavernous ICA, rather than a focal tear at the cavernous ICA seen in typical traumatic CCFs. The findings suggest that the severe ICA dissection likely harbors a false lumen with intimal entry at the proximal ICA and adventitial exit at the cavernous ICA. Therefore, it seems optimal to place self-expanding stents through the true lumen of the ICA along the dissecting segment to eliminate the false channel between the ICA and CS. Our aim is to investigate the causal relationship between the ICA dissections and CCFs during hybrid ICAO surgery and to characterize technical outcomes after treatment with stenting.

## Methods

Between December 2015 and June 2018, 5 patients with new development of intraprocedural CCF and severe ICA dissection after surgical removal of ICAO plaque underwent treatment with self-expanding stenting. We prospectively collected patients’ data and follow-up information. All patients signed the written informed consent, and the study was approved by the institutional ethics committee of the PLA Rocket Force Characteristic Medical Center (KY2013031).

### Indications and contraindications of hybrid surgery

Indications for hybrid surgery of symptomatic chronic ICAO were transient ischemic attack (TIA) or ischemic stroke within 3 months; total ICAO related to the cerebral ischemic event and documented by ultrasound, CT angiography (CTA), magnetic resonance angiography, or catheter angiography at least 2 weeks before the revascularization procedure; patency of the ipsilateral middle cerebral artery (MCA) via the Willis collaterals, or the ipsilateral ophthalmic artery or other external carotid-ICA collaterals; ipsilateral cerebral hypoperfusion revealed by CT or MR perfusion imaging; infarct size less than one third of the ipsilateral MCA territory on MR/CT images; and preprocedural treatment with aspirin 300 mg and clopidogrel 75 mg daily for at least 5 days.

Indications for stenting were concomitant spiral and diffuse dissection from the cervical to cavernous ICA and CCF on angiography after surgical removal of ICA plaque, no manipulation with Fogarty embolectomy catheter, and no evidence of CCF on preprocedural catheter angiography.

Contraindications were any bleeding disorder and allergies to heparin, aspirin, clopidogrel, iodine contrast, or general anesthesia.

### Procedures of CEA, angiography, and stenting

Initial CEA was performed at a neurovascular hybrid operating room (HOR) equipped with an Artis Zeego angiographic system (Siemens AG, Forchheim, Germany). The main procedural steps were as follows: supine position with the head turned to the contralateral side; general anesthesia; skin incision along the anterior border of the sternocleidomastoid muscle; exposure of the common carotid artery (CCA), ICA, and external carotid artery and then tightly looping each artery with elastic tourniquets; arteriotomy at the carotid bifurcation; dissection of the proximally occluded plaque exposed in the arteriotomy; removal of the plaque and organized thrombus linked with the distal end of the plaque; loosening the ICA tourniquet to observe blood backflow, followed by placement of a 5F sheath into the ICA at 1–2 cm distal to the ICA tourniquet for angiography of distal ICAO.

The femoral artery route was used for stenting procedure. After a 6F or 8F guiding catheter was advanced into the ICA arteriotomy site, an assembly of a Prowler Select Plus microcatheter (Codman & Shurtleff, Raynham, MA) and 0.014-in. Synchro exchange microwire (Stryker Neurovascular, Fremont, CA) was placed into the ICA at 1–2 cm distal to the ICA tourniquet. Angiography was then performed via the microcatheter. The assembly of microcatheter and microwire was used to find the true lumen of the ICA along the dissection segment and navigated into the normal ICA distal to the dissection under road map guidance. A self-expanding Wallstent (Boston Scientific Corporation, Natick, MA) was then deployed in the cervical ICA. The microcatheter was then exchanged into the normal ICA distal to the dissection, followed by deployment of one or more self-expanding Enterprise stents (Codman & Shurtleff, Raynham, MA) into the intracranial ICA and distal cervical ICA. The self-expanding stents were telescopically placed through true channels of the dissections forming a “stent-autograft” construct with the use of native in situ dissection flaps as stent-covering membranes. The arteriotomy was then closed before ending the hybrid surgery.

### Evaluation of pre-, intra-, and post-procedural angiographies and clinical follow-up

Pre-, intra-, and post-procedural angiographies were assessed with consensus by two physicians. Pre-procedural angiography was used to determine ICA occlusion length, appearance of the residual root proximal to the occlusion, and collateral circulation. ICAOs were classified into 4 categories: Type A or B, short occlusion less than 3/4 of the cervical ICA with or without a tapered residual root, respectively; and C or D, long occlusion with or without tapered residual root, respectively [12].

Intra-procedural angiography was used to categorize dissections as per the National Heart, Lung, and Blood Institute classification system [13] to determine CCF flow status, drainage pattern, and evidence of cortical venous reflux, as well as procedural success by the efficacy of ICAO recanalization and CCF obliteration. High-flow CCF was defined as rapid CS filling without filling of intracranial vessels, intermediate-flow CCF as rapid CS filling with filling of intracranial vessels, and low-flow CCF as slow and sluggish CS filling with filling of intracranial vessels [4]. Drainage patterns were categorized into anterior, posterior, and inferior ones, such as via the superior ophthalmic vein, via the inferior petrosal sinus and pterygoid venous plexus, respectively, or both. Successful recanalization of ICAO was defined as the restoration of Thrombolysis in Cerebral Infarction (TICI) 3 flow in the ICA and its distal branches and the complete or partial obliteration of CCF as no filling of CS or obvious reduction of the shunt flow, respectively, with filling of intracranial vessels.

Follow-up catheter angiography or CTA was used to assess status of ICA patency and CCF obliteration. Patients were followed up daily during hospitalization and in the clinic or by telephone after hospital discharge until the end of December 2018. Data on stroke and CCF syndrome were collected during hospitalization and at follow-up.

## Results

Between January 2014 and July 2018, a total of 98 hybrid procedures were performed for consecutively enrolled ICAO patients, 18 of which developed CCFs related to the procedure (18.4%, 18/98). Five of the latter were successfully treated. Successful recanalization was finally achieved in 82 of 98 ICAO patients (84%). All 5 patients were male with an average age of 66.2 ± 5.4 years (Table 1). Their CCFs were associated with spiral (type D) and diffuse dissection from the cervical to cavernous ICA, with high-flow CCF in 2 patients, intermediate-flow CCF in 1, and low-flow CCF in 2 and with inferior drainage in 2, posterior drainage in 1, posterior and inter-cavernous drainage in 1, and anterior and inferior drainage in 1. No patient had evidence of cortical venous reflux.

**Table 1:** Baseline and procedural data and outcomes of treatment with stenting in 5 patients

No.	Gender/age	Risk factors	Occlusion side	Qualified events	Collaterals	Duration^†^ (days)	CCF flow^‡^	CCF drainage	ET and stents^§^	Seal of CCF/CCF syndrome/recanalization of ICA	Clinical FU (month)/event/CCF syndrome	Angiography FU (month)/complete CCF seal/ICA patency
1	M/60	DM, HL, HT	R	TIA	PCoA	20	Low	Inferior	EP 4.5 × 37 mm + Wallstent 7 × 50 mm	Partial/pulsatile tinnitus/yes	37/no/no	24/yes/yes
2	M/68	HL	R	Stroke	PCoA	210	Low	Inferior	EP 4.5 × 37 mm + Wallstent 9 × 50 mm	Complete/no/yes	14/no/no	12/yes/yes
3	M/61	CS, HL, HT	L	Stroke	PCoA	14	High	Inter-cavernous, posterior	EP 4.5 × 37 mm*2 + Wallstent 7 × 50 mm	Complete/no/yes	16/no/no	12/yes/yes
4	M/70	CS, HT	L	TIA	ACoA, Oph	16	Intermediate	Anterior, inferior	EP 4.5 × 37 mm*2 + Wallstent 7 × 40 mm	Complete/no/yes	6/no/no	6/yes/yes
5	M/72	CS, HL	L	Stroke	ACoA, PCoA, Oph	43	High	Posterior	EP 4.5 × 37 mm*2 + Wallstent 7 × 40 mm	Complete/no/yes	9/no/no	6/yes/no

*ACoA* anterior communicating artery, *CS* cigarette smoking, *DM* diabetes mellitus, *FU* follow-up, *HL* hyperlipidemia, *HT* hypertension, *L* left, *M* male, *OAO* ipsilateral ophthalmic artery or other external carotid-ICA collaterals, *PCoA* posterior communicating artery, *R* right, *TIA* transient ischemic attack

^†^Days from ICAO documentation to procedure

^‡^High-flow CCF, rapid CS filling without filling of intracranial vessels; intermediate-flow CCF, rapid CS filling with filling of intracranial vessels; low-flow CCF, slow and sluggish CS filling with filling of intracranial vessels

^§^*ET* endovascular therapy, *EP* Enterprise stents (Codman & Shurtleff, Raynham, MA), *WS* Wallstent (Boston Scientific Corporation, Natick, MA)

Pulsatile tinnitus resolved 1 year later

**Fig. 1:**
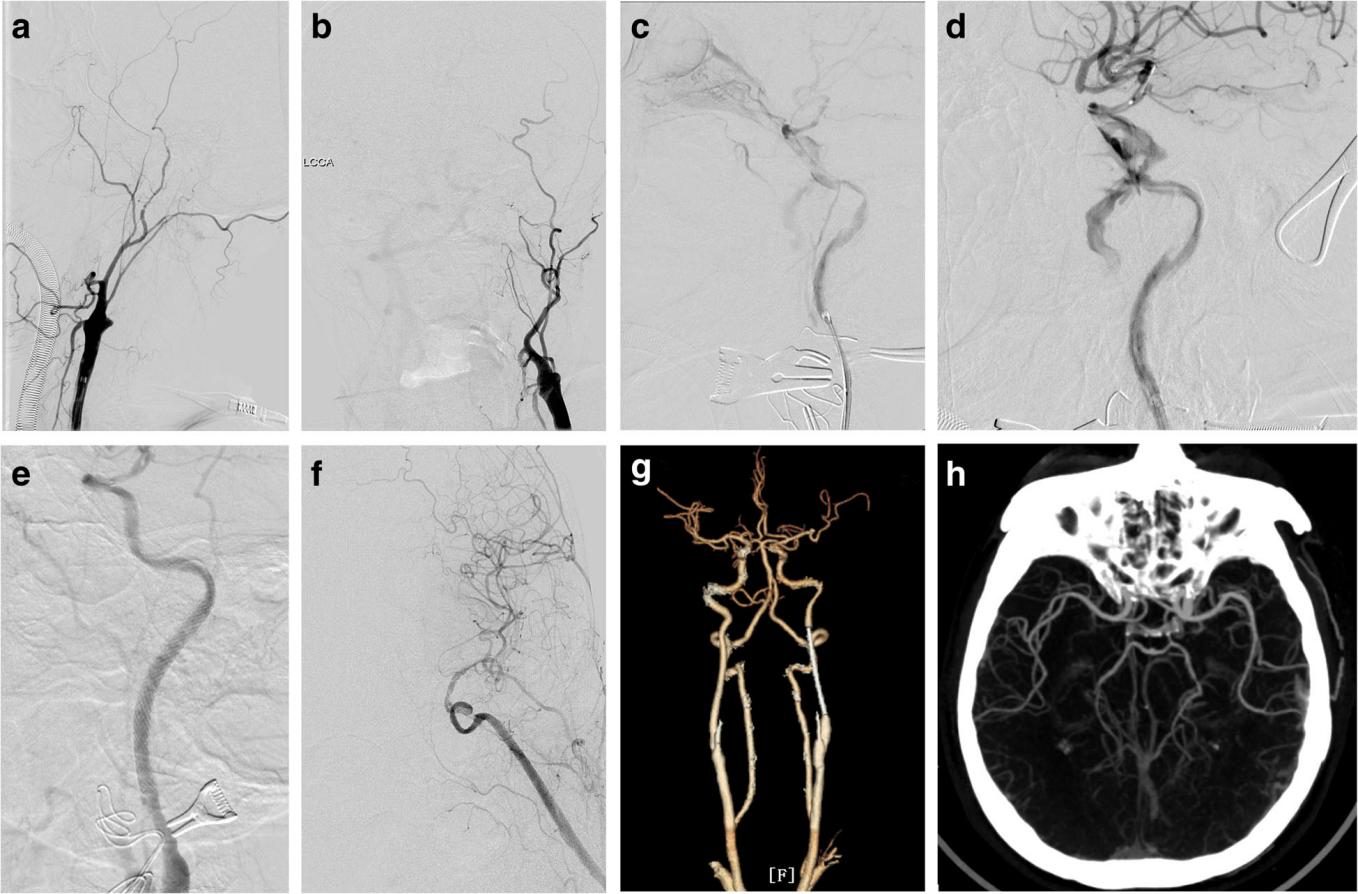
Case example. Case 4 of hybrid surgery for a symptomatic chronic internal carotid artery occlusion (ICAO) and a new development of intraprocedural carotid-cavernous fistula (CCF). **a**, **b** Left ICAO, revealed by preoperative angiographies on lateral and posterior-anterior projection, respectively. **c** A new development of intermediate-flow CCF with anterior and inferior drainage, along with spiral and cervical-to-cavernous ICA dissection after surgical removal of the ICAO plaque, revealed by angiographies via PLUS microcatheter (Codman & Shurtleff, Raynham, MA). **d** Angiography via the guiding catheter following negotiation of the assembly of microcatheter and microwire into normal ICA distal to the dissection. **e**, **f** Successful ICA recanalization and complete CCF obliteration after telescopically deploying one 7 × 40 mm Wallstent (Boston Scientific Corporation, Natick, MA) and two 4.5 × 37 mm Enterprise stents through the true channel of the dissection. **g**, **h** Durable patency of the left ICA and complete CCF obliteration on follow-up CT angiogram at 6 months

The assembly of microcatheter and microwire was successfully navigated through the true lumen of the ICA dissection into the distal normal ICA in all patients. A total of 13 stents were placed across the dissection with an average of 2.6 stents (range, 2 to 3 stents) per patient, including 5 Wallstent (1 per patient) and 8 Enterprise stents (1 to 2 each one). After stenting, successful dissection reconstruction with TICI 3 flow was achieved in all patients, with complete and partial obliteration of CCF in 4 and 1, respectively (Fig. 1).

No patient had CCF syndrome and stroke or death during follow-up of 6 to 37 months. However, one patient (case 1) developed pulsatile tinnitus, which resolved 1 year later. Angiography at 6 to 24 months showed complete CCF obliteration in all 5 patients and ICA patency with TICI 3 flow restoration in 4 patients.

## Discussion

This study showed CCF formation along with spiral and diffuse dissection of the cervical-to-cavernous ICA during ICAO surgery. The mechanism of CCF formation is likely dissection-related because of the successful treatment of CCFs with self-expanding stenting through the true channel of dissection. Our results suggest that utilizing native in situ dissection flap as covering membrane of a stent may be an optimal solution for the CCF complication from ICAO surgery, with favorable safety and efficacy. In this series, the stenting alone immediately recanalized all 5 dissections post ICAO surgery and simultaneously obliterated 4 CCFs completely and 1 partially, without periprocedural stroke or death, and achieved CCF obliteration in all 5 patients and ICA patency in 4 patients on follow-up angiography of 6 to 24 months.

**Fig. 2:**
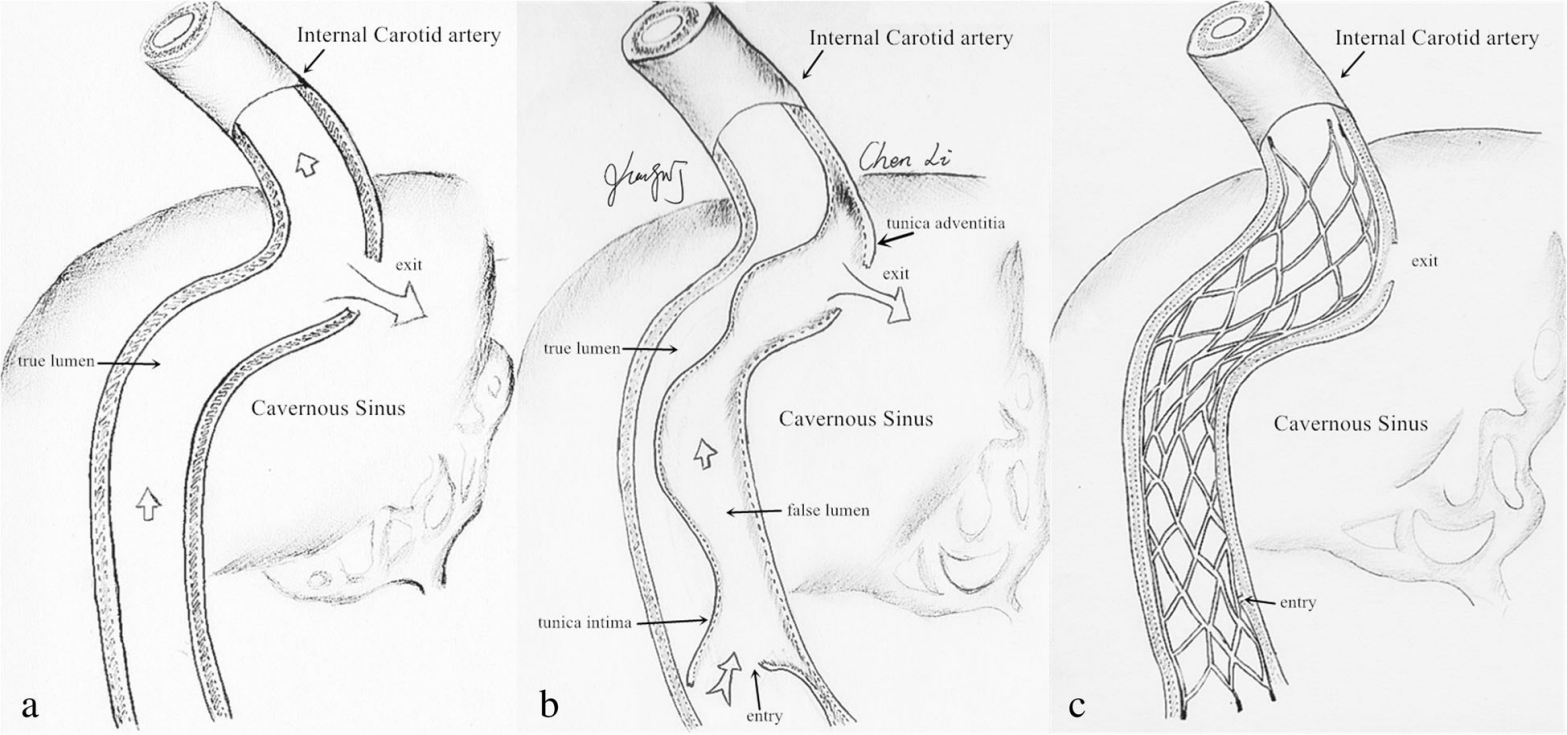
Disparities in anatomy between the novel CCFs and the typical traumatic CCFs. **a** The typical traumatic CCF has an intima-to-adventitia tear between the cavernous internal carotid artery (ICA) and the cavernous sinus (CS). **b** The novel CCF is composed of the ICA lumen, intimal entry, false channel, adventitial exit, and CS. **c** The concept of “self-expanding stent graft” means the use of native in situ dissection flap as covering membrane of the self-expanding stent to reconstruct true channel of the spiral dissection and simultaneously obliterate the novel CCF

The successful dissection reconstruction and CCF obliteration after stenting strongly support our previous hypothesis that the severe ICA dissection likely harbors a false channel with intimal entry at the proximal ICA and adventitial exit at the cavernous ICA. Self-expanding stenting via true lumen can obliterate CCF by eliminating the false channel of the dissection. The native in situ dissection flap is used as covering membrane of the self-expanding stent to reconstruct the true channel of the spiral dissection and simultaneously obliterate the atypical CCF. Apparently, there is disparity in the anatomical component between the atypical dissection-associated CCFs and the traditional traumatic CCFs. The former consists of the ICA lumen, intimal entry, false channel, adventitial exit, and CS, while the latter only has an intima-to-adventitia opening to connect the cavernous ICA and CS (Fig. 2). Stenting seems to be feasible, safe, and effective for both ICA dissection reconstruction and CCF obliteration rather than other treatment options. ICA ligation may be simple and effective for the treatment of intraprocedural CCFs but may lead to the ICAO recanalization failure. Stenting cannot be extrapolated to be the treatment choice of other iatrogenic CCFs. The efficacy of placing self-expanding stents or flow diverters (FDs) for other traumatic CCFs remains uncertain [6–8].

The spiral and long segmental dissection after surgical treatment of chronic ICAO may be secondary to the intraprocedural injury to the ICA wall that had ischemic injury due to chronic ICA occlusion. Intra-arterial manipulations, such as manually tugging an organized thrombus distal to arteriotomy, inserting a neck sheath via ICA arteriotomy, and even angiography in the false lumen, may have caused initial dissection and further propagation. It is necessary to continuously improve the operating procedures of the hybrid surgery for chronic ICAO. The following modifications may be helpful to alleviate injury to the ICA: use of ring stripping for a distally organized thrombus rather than manually tugging [14], placement of a microcatheter into the occluded ICA distal to arteriotomy after surgical removal of proximal occlusion rather than neck sheath, angiography in the dissection segment via microcatheter rather than conventional catheter or sheath.

Injury-repair response of vessel wall to hypoxia may differ as time lapses. In the circumstance of chronic occlusion, vessel adventitia can act as a biological central processing unit in vessel wall function, likely from harboring a wide variety of components such as fibroblasts, inflammatory cells, stem/progenitor cells, and vasa vasorum (VV, microvessels of a vessel). Therefore, oxygen and nutrients to the inner wall of a vessel in the occluded segment may be completely supplied by adventitial VV. In response to injury, VV conduits proliferate in adventitia and extend to intima by interacting with vascular resident stem cells (VSCs) housed in the VV niche. Angiogenesis of VSCs may be beneficial for vasculature and plaque stabilization, when VSCs supplied and transported by VV enter into intima and differentiate into endothelial cells, pericytes, and fibroblasts and when pericytes interact with endothelial cells enough. However, neovessels of VV are immature with a characteristically thin wall with less pericyte encirclement. The fragile structure may lead to microvascular cleft that is responsible for leakage of red blood cells (intramural hemorrhage) and accumulation of inflammatory cells (chronic inflammation). Additionally, VSCs can also differentiate into vascular smooth muscle cells associated with acceleration of plaque growth and reduction of vascular stability. The above-described unfavorable responses may increase vulnerability of the intimal layer of non-acute ICAO to mechanical injury, especially when operating in atherosclerotic vessels. Normalization of the impaired vasa vasorum would be an attractive therapeutic strategy; however, this is not yet available in clinical practice [15]. Of note, the cavernous ICA exit site for the false channel dissection in our patients could be partially explained by the concurrent location of the atretic atrophy of the primitive trigeminal artery, potentially resulting in vessel wall weakening [1].

## Limitations

This study reports our experience in the management of atypical CCFs with long and spiral ICA dissection post initial CEA for chronic ICAOs in the hybrid OR with telescoping self-expanding stents. Despite the novelty of our report, our case series is very small with only 5 patients and our preliminary data needs to be confirmed in a larger sample size. Nevertheless, the atypical CCF may be encountered more often with increasing application of hybrid surgery for in situ reconstruction of symptomatic chronic ICAOs. It is beneficial for neurointerventionalists to be aware of the possibilities of atypical CCFs and various treatment options.

## Conclusions

The carotid-cavernous fistulas (CCFs) with spiral and long segmental dissection from the cervical-to-cavernous ICA during surgery of chronic ICAOs are dissection-related because of successful obliteration after stenting for dissection reconstruction. Self-expanding stenting through true channel of the dissection, serving as implanting stent-autograft, may be an optimal therapy for the atypical CCF complication from ICAO surgery.

## Data Availability

The datasets used and/or analyzed during the current study are available from the corresponding author on reasonable request.

## Abbreviations

CCF: Carotid-cavernous fistula
CEA: Carotid endarterectomy
CS: Cavernous sinus
CTA: Computed tomography angiography
ICAO: Internal carotid artery occlusion
TIA: Transient ischemic attack
VSCs: Vascular resident stem cells
VV: Vasa vasorum

## References

[1] Helmke K, Krüger O, Laas R (1994). The direct carotid cavernous fistula: a clinical, pathoanatomical, and physical study. Acta Neurochir.

[2] Ellis JA, Goldstein H, Connolly ES (2012). Carotid-cavernous fistulas. Neurosurg Focus.

[3] Henderson A D, Miller N R (2017). Carotid-cavernous fistula: current concepts in aetiology, investigation, and management. Eye.

[4] van Rooij WJ, Sluzewski M, Beute GN (2006). Ruptured cavernous sinus aneurysms causing carotid cavernous fistula: incidence, clinical presentation, treatment, and outcome. Am J Neuroradiol.

[5] Chi CT, Nguyen D, Duc VT (2014). Direct traumatic carotid cavernous fistula: angiographic classification and treatment strategies. Study of 172 cases. Interv Neuroradiol.

[6] Nadarajah M, Power M, Barry B, Wenderoth J (2012). Treatment of a traumatic carotid-cavernous fistula by the sole use of a flow diverting stent. J Neurointerv Surg.

[7] Pradeep N, Nottingham R, Kam A (2016). Treatment of post-traumatic carotid-cavernous fistulas using pipeline embolization device assistance. J Neurointerv Surg.

[8] Wendl CM, Henkes H, Moreno RM (2017). Direct carotid cavernous sinus fistulae: vessel reconstruction using flow-diverting implants. Clin Neuroradiol.

[9] Alan N, Nwachuku E, Jovin TJ (2017). Management of iatrogenic direct carotid cavernous fistula occurring during endovascular treatment of stroke. World Neurosurg.

[10] Wang W, Li MH, Li YD (2016). Reconstruction of the internal carotid artery after treatment of complex traumatic direct carotid-cavernous fistulas with the Willis covered stent: a retrospective study with long-term follow-up. Neurosurgery.

[11] Shih YT, Chen WH, Lee WL (2013). Hybrid surgery for symptomatic chronic total occlusion of carotid artery: a technical note. Neurosurgery.

[12] Jiang WJ, Liu AF, Yu W (2019). Outcomes of multimodality in-situ recanalization in hybrid operating room (MIRHOR) for symptomatic chronic internal carotid artery occlusions. J Neurointerv Surg.

[13] Huber MS, Mooney JF, Madison J, Mooney MR (1991). Use of a morphologic classification to predict clinical outcome after dissection from coronary angioplasty. Am J Cardiol.

[14] Pinter L, Cagiannos C, Bakoyiannis CN (2007). Hybrid treatment of common carotid artery occlusion with ring-stripper endarterectomy plus stenting. J Vasc Surg.

[15] Kawabe Jun-ichi, Hasebe Naoyuki (2014). Role of the Vasa Vasorum and Vascular Resident Stem Cells in Atherosclerosis. BioMed Research International.

